# 84. Improving Traditional Registrational Trial Endpoints: Development and Application of a Desirability of Outcome Ranking (DOOR) Endpoint for Acute Bacterial Skin and Skin-Structure Infections

**DOI:** 10.1093/ofid/ofae631.021

**Published:** 2025-01-29

**Authors:** Jessica Howard-Anderson, Toshimitsu Hamasaki, Lizhao Ge, Deborah Collyar, Sumathi Nambiar, Carol Hill, Holly S Geres, Sara E Cosgrove, Thomas L Holland, Sarah B Doernberg, Henry Chambers, Vance G Fowler, Scott R Evans, Helen Boucher

**Affiliations:** Emory University, Atlanta, GA; The George Washington University, Rockville, MD; George Washington University, Rockville, Maryland; Patient Advocates In Research (PAIR), Danville, California; Johnson and Johnson, Germantown, Maryland; Duke Clinical Research Institute, Durham, North Carolina; Duke Clinical Research Institute, Durham, North Carolina; Johns Hopkins School of Medicine, Baltimore, MD; Duke University Medical Center, Durham, NC; University of California, San Francisco, San Francisco, CA; University of California San Francisco, San Francisco, California; Duke University Medical Center, Durham, NC; Milken Institute School of Public Health, Rockville, MD; Tufts University School of Medicine, Boston, MA

## Abstract

**Background:**

The ideal primary endpoint for acute bacterial skin and skin-structure infection (ABSSSI) trials is unknown and regulatory guidance differs between U.S. and Europe. Desirability of outcome ranking (DOOR) is a paradigm for the design, analysis and interpretation of clinical trials that incorporates a benefit-risk evaluation and provides a global assessment of patient experience.

**Figure:**
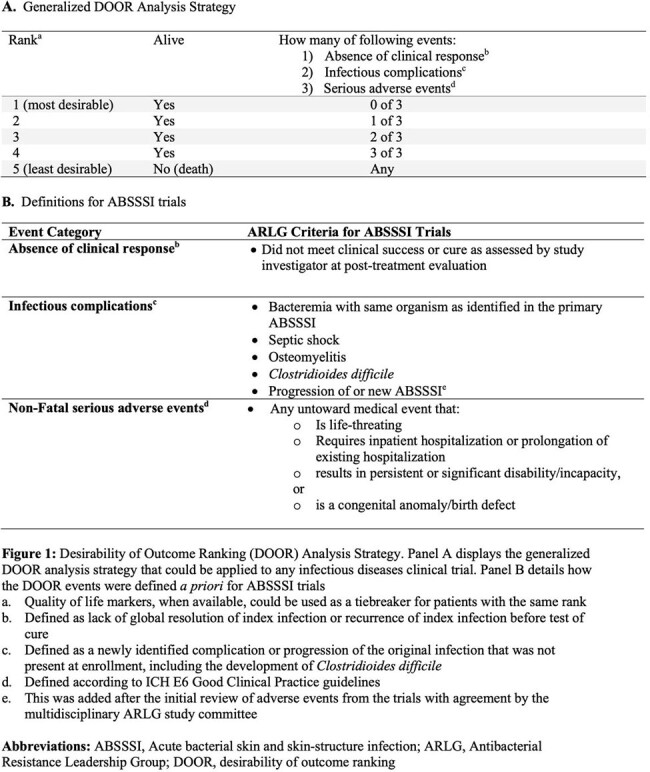

**Methods:**

Drawing on prior work from the Antibacterial Resistance Leadership Group (ARLG), a multidisciplinary committee of experts in infectious diseases, clinical trial design, drug regulation, and patient experience adapted a generalized infectious diseases DOOR for ABSSSI trials (Figure 1). We then retrospectively applied this to two previously published, noninferiority, double-blind, randomized, registrational, ABSSSI trials comparing omadacycline to linezolid OASIS-1 and OASIS-2, Figure 2) to demonstrate how DOOR could be applied. We compared the DOOR distribution and probability of having a more desirable outcome between treatment groups and performed DOOR component analyses.

**Figure:**
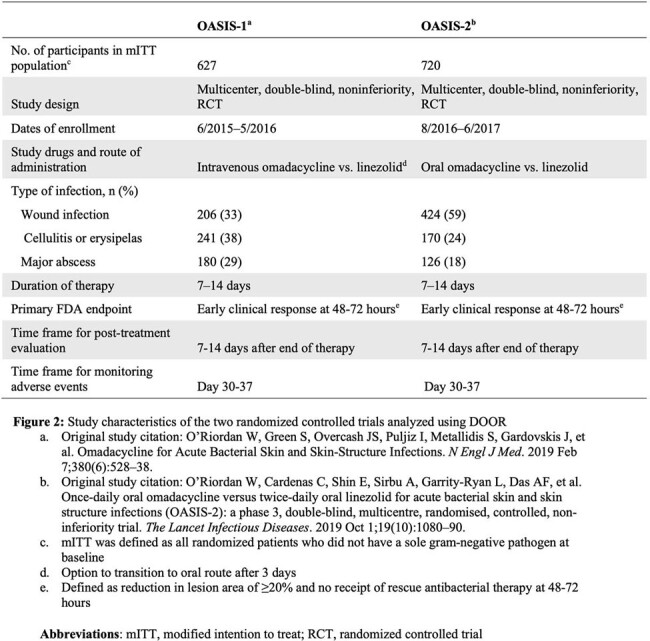

**Results:**

For both trials, the ABSSSI DOOR demonstrated similar overall clinical outcomes between treatment groups. In OASIS-1, the probability that a participant treated with omadacycline would have a more desirable outcome than a participant treated with linezolid was 50.7% (95% confidence interval [CI], 47.7%−53.8%) (Figure 3). In OASIS-2, the probability that a participant treated with omadacycline would have a more desirable outcome than a participant treated with linezolid was 51.0% (95% CI, 48.1%–53.8%) (Figure 4). Overall, the DOOR components (clinical failure, infectious complications, serious adverse events, and death) were rare, although the DOOR component analysis provided a more in-depth understanding of the potential trade-offs between efficacy and safety when comparing omadacycline to linezolid.

**Figure:**
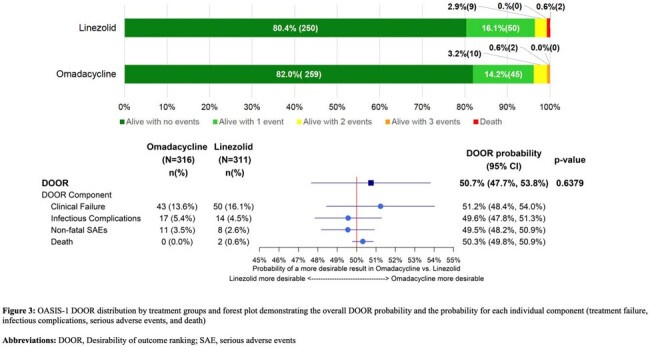

**Conclusion:**

DOOR can be informative in registrational ABSSSI trials. Using DOOR can provide clinicians with a better understanding of the clinical outcomes of patients with skin infections. Future trials using DOOR prospectively can continue to inform how to best use DOOR in ABSSSI trials.

**Figure:**
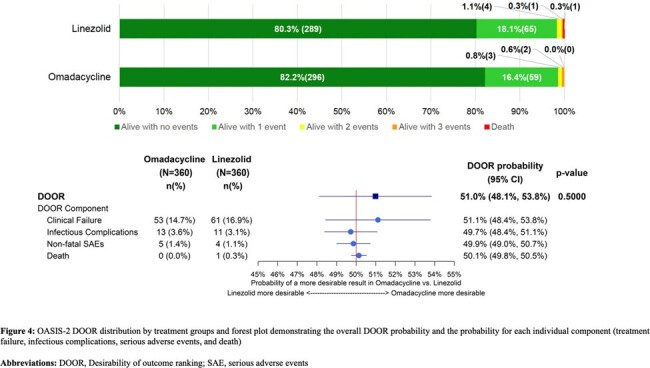

**Disclosures:**

**Deborah Collyar, B.Sci**, Apellis Pharmaceuticals, Inc.: Advisor/Consultant|Kinnate Biopharma: Advisor/Consultant **Sumathi Nambiar, MD MPH**, Johnson and Johnson: Stocks/Bonds (Public Company) **Carol Hill, PhD**, Glaxo SmithKline: Retirement Health, Cash Balance Plan|Glaxo SmithKline: Stocks/Bonds (Public Company) **Thomas L. Holland, MD**, Karius, Inc: Advisor/Consultant **Sarah B. Doernberg, MD, MAS**, Basilea Pharmaceutica: Grant/Research Support|F2G Limited: Grant/Research Support|Genentech: Advisor/Consultant|Gilead Biosciences: Grant/Research Support|Janssen/J+J: Advisor/Consultant|Pfizer, Inc: Grant/Research Support|Regeneron, Inc: Grant/Research Support|Shinogi: Grant/Research Support **Henry Chambers, MD**, Merck: Stocks/Bonds (Private Company)|Moderna: Stocks/Bonds (Private Company) **Vance G. Fowler, MD, MHS**, Affinergy: Advisor/Consultant|ArcBio: Stocks/Bonds (Private Company)|Armata: Advisor/Consultant|Astra Zeneca: Advisor/Consultant|Astra Zeneca: Grant/Research Support|Basilea: Advisor/Consultant|Basilea: Grant/Research Support|ContraFect: Advisor/Consultant|ContraFect: Grant/Research Support|Debiopharm: Advisor/Consultant|Destiny: Advisor/Consultant|EDE: Grant/Research Support|Genentech: Advisor/Consultant|Genentech: Grant/Research Support|GSK: Advisor/Consultant|Janssen: Advisor/Consultant|Karius: Grant/Research Support|MedImmune: Grant/Research Support|Merck: Grant/Research Support|sepsis diagnostics: Patent pending|UptoDate: Royalties|Valanbuio: Stocks/Bonds (Private Company)|Valanbuio: Stocks/Bonds (Private Company) **Scott R. Evans, PhD, M.S.**, Abbvie: Advisor/Consultant|Advantagene: Advisor/Consultant|Akouos: Advisor/Consultant|Apellis: Advisor/Consultant|AstraZenenca: Advisor/Consultant|BARDA: Advisor/Consultant|Breast International Group: Advisor/Consultant|CDC: Grant/Research Support|Clover: Advisor/Consultant|DayOneBio: Advisor/Consultant|Degruyter: Editorial service|Duke University: Advisor/Consultant|Eli Lilly: Advisor/Consultant|FDA: Advisor/Consultant|Frontier Science Foundation: Board Member|Genentech: Advisor/Consultant|GSK: Advisor/Consultant|Henry Jackson Foundation: Grant/Research Support|International Drug Development Institute: Advisor/Consultant|Johson & Johnson: Advisor/Consultant|Medtronic: Advisor/Consultant|NIH: Grant/Research Support|Novartis: Advisor/Consultant|Pfizer: Advisor/Consultant|Rakuten: Advisor/Consultant|Roche: Advisor/Consultant|Takeda: Advisor/Consultant|Taylor & Francis: Book royalties|Teva: Advisor/Consultant|University of Penn: Advisor/Consultant|Vir: Advisor/Consultant|Wake Forest University: Advisor/Consultant|Washington University: Advisor/Consultant **Helen Boucher, MD**, ASM: Honoraria|Elsevier: Honoraria|Sanford Guide: Honoraria

